# Hypoxia-inducible factor-1α is the therapeutic target of the SGLT2 inhibitor for diabetic nephropathy

**DOI:** 10.1038/s41598-019-51343-1

**Published:** 2019-10-14

**Authors:** Ryoichi Bessho, Yumi Takiyama, Takao Takiyama, Hiroya Kitsunai, Yasutaka Takeda, Hidemitsu Sakagami, Tsuguhito Ota

**Affiliations:** Division of Metabolism and Biosystemic Science, Department of Internal Medicine, Asahikawa Medical University, 2-1-1-1 Midorigaoka Higashi, Asahikawa, 078-8510 Japan

**Keywords:** Diabetes complications, Chronic kidney disease

## Abstract

Previous studies have demonstrated intrarenal hypoxia in patients with diabetes. Hypoxia-inducible factor (HIF)-1 plays an important role in hypoxia-induced tubulointerstitial fibrosis. Recent clinical trials have confirmed the renoprotective action of SGLT2 inhibitors in diabetic nephropathy. We explored the effects of an SGLT2 inhibitor, luseogliflozin on HIF-1α expression in human renal proximal tubular epithelial cells (HRPTECs). Luseogliflozin significantly inhibited hypoxia-induced HIF-1α protein expression in HRPTECs. In addition, luseogliflozin inhibited hypoxia-induced the expression of the HIF-1α target genes *PAI-1*, *VEGF*, *GLUT1*, *HK2* and *PKM*. Although luseogliflozin increased phosphorylated-AMP-activated protein kinase α (p-AMPKα) levels, the AMPK activator AICAR did not changed hypoxia-induced HIF-1α expression. Luseogliflozin suppressed the oxygen consumption rate in HRPTECs, and subsequently decreased hypoxia-sensitive dye, pimonidazole staining under hypoxia, suggesting that luseogliflozin promoted the degradation of HIF-1α protein by redistribution of intracellular oxygen. To confirm the inhibitory effect of luseogliflozin on hypoxia-induced HIF-1α protein *in vivo*, we treated male diabetic *db/db* mice with luseogliflozin for 8 to 16 weeks. Luseogliflozin attenuated cortical tubular HIF-1α expression, tubular injury and interstitial fibronectin in *db/db* mice. Together, luseogliflozin inhibits hypoxia-induced HIF-1α accumulation by suppressing mitochondrial oxygen consumption. The SGLT2 inhibitors may protect diabetic kidneys by therapeutically targeting HIF-1α protein.

## Introduction

Diabetic nephropathy is the most common disease resulting in end-stage renal disease (ESRD)^1^ and therefore it is imperative to develop an effective treatment for diabetic nephropathy.

Sodium-glucose cotransporter 2 (SGLT2) inhibitors, a novel class of antidiabetic medications, target the renal proximal tubules to reduce glucose reabsorption, leading to increased urinary glucose excretion and anti-hyperglycemic effects. Recent clinical trials have demonstrated the renoprotective effects of SGLT2 inhibitors in diabetic nephropathy^2–5^. SGLT2 inhibitors are now recommended as a second-line medication for patients with atherosclerotic cardiovascular disease or chronic kidney diseases for the management of type 2 diabetes^6^. However, the mechanisms of how SGLT2 inhibitors prevent diabetic nephropathy, especially their direct effect on proximal tubular cells, have not been fully elucidated.

Hypoxia status of renal tubular cells is known to cause fibrosis in diabetic kidney^7^. A key molecule that plays an important role in hypoxic conditions is hypoxia-inducible factor (HIF)-1α. HIF-1 is a heterodimeric transcription factor composed of an oxygen-sensitive α subunit and a constitutively expressed β subunit^8,9^. The transcriptional activity of HIF-1 is minutely regulated by the stability of HIF-1α protein, which is quickly degraded via ubiquitin-proteasome pathway under normoxic condition. Hypoxia in renal tubules has been considered as a common feature of early and advanced stages of diabetic nephropathy^10,11^. In addition, diabetes increased HIF-1ααexpression in proximal tubular cells in a type 2 diabetic animal model with nephropathy^12,13^ and in renal tissues from patients with diabetic nephropathy^14^.

Stable HIF-1α expression in tubular epithelial cells leads to tubulointerstitial fibrosis^15–18^. In addition, plasminogen activator inhibitor-1 (PAI-1), a major HIF-1 target gene, is also an important factor for the progression of kidney fibrosis, and previous studies showed that genetically silencing *Pai-1* alleviates diabetic nephropathy in mice^19,20^. On the other hand, the pharmacological inhibition of HIF-1α by an HIF-1 inhibitor (YC-1;3-(5′-hydroxymethyl-2′-furyl)-1-benzyl indazole), improved kidney fibrosis in type 1 diabetic OVE26 mice^21^. Thus, HIF-1 represents a potential candidate for the therapeutic interventions for diabetic nephropathy. However there is no clinical treatment targeting renal hypoxia in diabetic nephropathy to date.

These findings led us to study the renoprotective effects of the SGLT2 inhibitor luseogliflozin from the point of view of its impacts on renal hypoxia and HIF-1 α expression in human renal proximal tubular epithelial cells (HRPTECs) and proximal tubules in type 2 diabetes model *db/db* mice.

## Results

### Luseogliflozin inhibits hypoxia-induced HIF-1α protein expression

**Figure 1:**
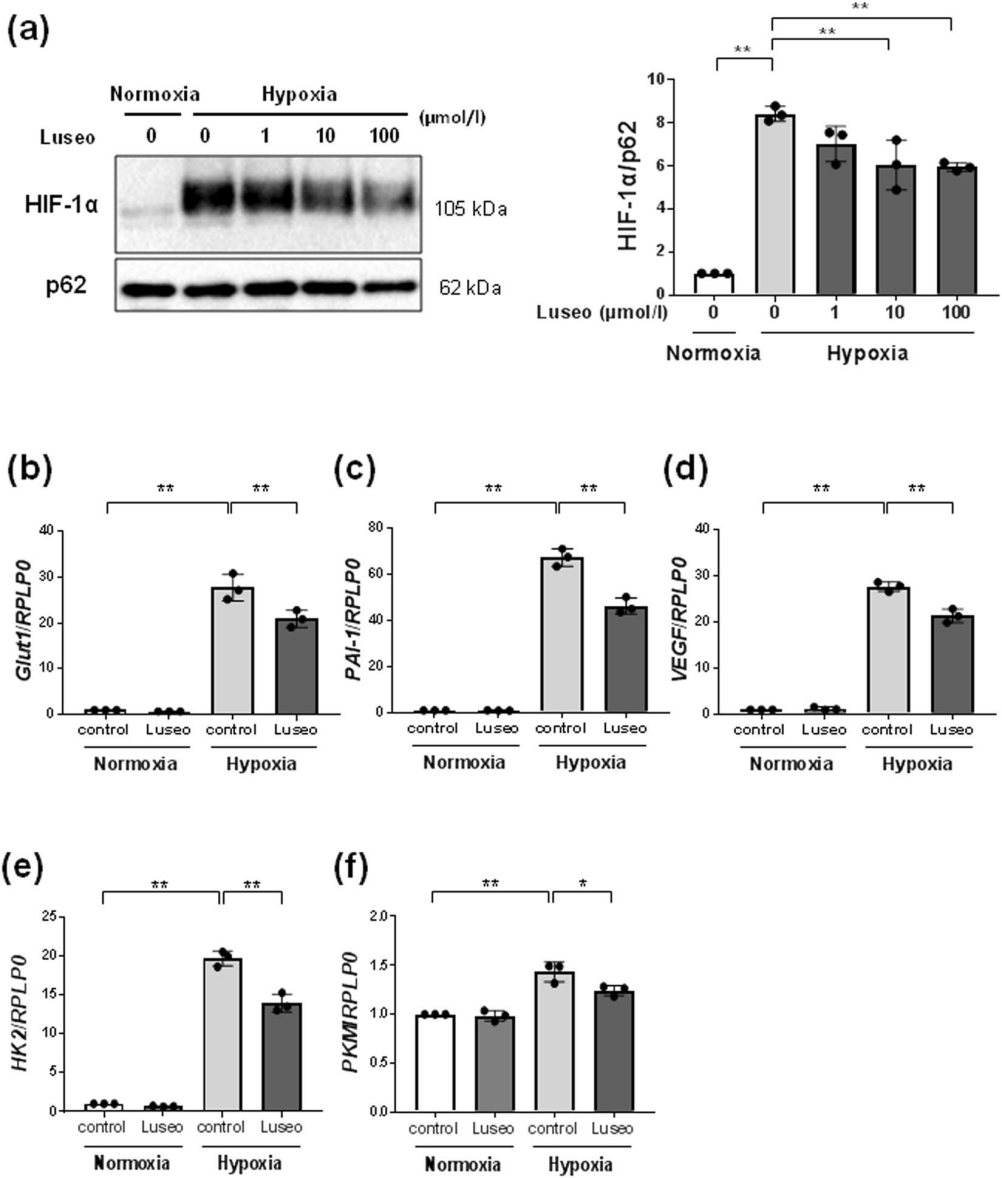
Effects of luseogliflozin on hypoxia-induced HIF-1α protein and HIF-1α target gene expression. (**a**) Luseogliflozin inhibits hypoxia-induced HIF-1α protein expression. HRPTECs were incubated in serum-free DMEM with 1–100 µmol/l luseogliflozin under normoxic (21% O_2_) or hypoxic (1% O_2_) conditions for 24 h. The protein expression of HIF-1α was determined by western blot analysis and quantified by densitometry, with p62 as the loading control (*n* = 3). All protein levels are expressed as fold of control. (**b**–**f**) Quantitative real-time RT-PCR analysis of HIF-1 target genes. HRPTECs were treated with or without 100 µmol/l luseogliflozin under normoxic and hypoxic conditions for 24 h. Total RNA was extracted from HRPTECs and used for quantitative RT-PCR (*n* = 3). The relative amounts of *GLUT 1*, *PAI-1*, *VEGF*, *HEK2* and *PKM* mRNA were normalized to *RPLP0* and expressed as an arbitrary unit in which the control group value equaled 1. All results are shown as the means ± SD. *p < 0.05, **p < 0.01, by one-way ANOVA followed by Tukey’s multiple comparison test.

HRPTECs faintly expressed HIF-1α protein under normoxic conditions (Fig. 1a). Hypoxia treatment (1% O_2_) markedly induced HIF-1α protein accumulation in HRPTECs, and luseogliflozin at 10–100 µmo/l significantly inhibited hypoxia-induced HIF-1α protein expression (Fig. 1a). Hypoxia-induced HIF-1α protein expression by more than 8-fold compared to the normoxic control condition (Supplementary Table 1, *p* < 0.01). Luseogliflozin (100 μmol/l) significantly decreased hypoxia-induced HIF-1α protein to 70.7 ± 2.3% (Supplementary Table 1, *p* < 0.01).

### Luseogliflozin inhibits HIF-1 target gene expression

We also examined the effects of luseogliflozin on the expression of HIF-1 target genes in HRPTECs (Fig. 1b–f). Quantitative RT-PCR results showed that hypoxia significantly promoted *GLUT1*, *PAI-1* and *VEGF* gene expression in HRPTECs (Supplementary Table 2, *p* < 0.01). Luseogliflozin (100 μmol/l) significantly reduced these hypoxia-induced mRNA expression levels (*p* < 0.01). In addition, luseogliflozin also inhibited the expression of hypoxia-induced hexokinase 2 (*HK2*)^22^, which catalyzes the first step of glucose metabolism, and pyruvate kinase M1/2 (*PKM*)^23^, a rate-limiting glycolytic enzyme (Fig. 1e,f).

### Luseogliflozin increases AMPK phosphorylation, and an AMPK activator and inhibitor do not affect HIF-1α protein expression

**Figure 2:**
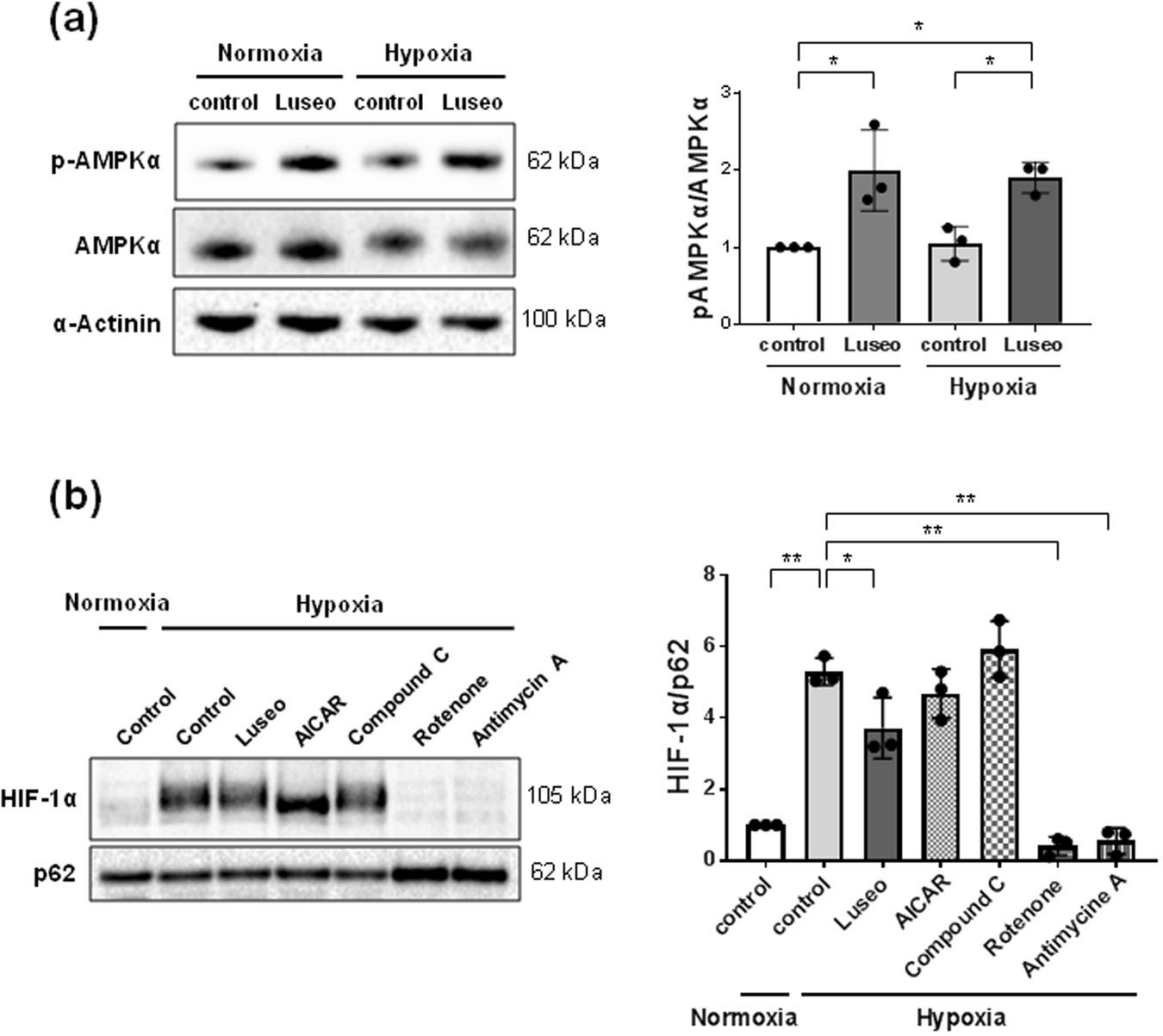
Luseogliflozin inhibits the hypoxia-induced HIF-1α protein, independent of AMPK activation. (**a**) Protein levels of pAMPKα were determined by western blot analysis and quantitated by densitometry (n = 3). HRPTECs were treated with 100 μmol/l luseogliflozin under normoxic or hypoxic conditions for 24 h. Then, total cellular extracts from HRPTECs were analyzed by western blot analysis and quantified by densitometry, with α-actinin as the loading control (n = 3). Luseogliflozin promoted the phosphorylation of AMPK under normoxia and hypoxia. (**b**) The inhibitors of mitochondrial respiratory complexes I and III, but not the AMPK activator and inhibitor, inhibited hypoxia-induced HIF-1α accumulation in HRPTECs. HRPTECs were treated with AICAR (1 mmol/l), compound C (20 µmol/l), rotenone (1 μmol/l) and antimycin A (10 ng/mL) under hypoxic conditions for 24 h. Nuclear extracts from HRPTECs were analyzed by western blot analysis and quantified by densitometry, with p62 as the loading control (n = 3). All results are shown as the means ± SD. *p < 0.05, **p < 0.01, by one-way ANOVA followed by Tukey’s multiple comparison test.

Luseogliflozin increased AMPKα phosphorylation (Th172) under normoxia and hypoxia by approximately 2-fold (*p* < 0.05) (Fig. 2a and Supplementary Table 3). However, AICAR, an AMPK activator, failed to suppress hypoxia-induced HIF-1α expression (Fig. 2b and Supplementary Table 4). In addition, an AMPK inhibitor, compound C (20 µmol/l), also failed to change HIF-1α protein expression (Fig. 2b and Supplementary Table 4).

### Mitochondrial inhibitors decrease hypoxia-induced HIF-1α protein expression

To determine the mechanism implicated in the regulation of HIF-1α expression in HRPTECs, subsequent experiments were performed using inhibitors of mitochondrial respiratory complex I (rotenone, 1 µmol/l) and mitochondrial respiratory complex III (antimycin A, 10 ng/ml). These inhibitors of mitochondrial respiration suppressed hypoxia-induced HIF-1α expression (*p* < 0.01) (Fig. 2b and Supplementary Table 4).

### Luseogliflozin decreases the OCR and intracellular ATP levels

**Figure 3:**
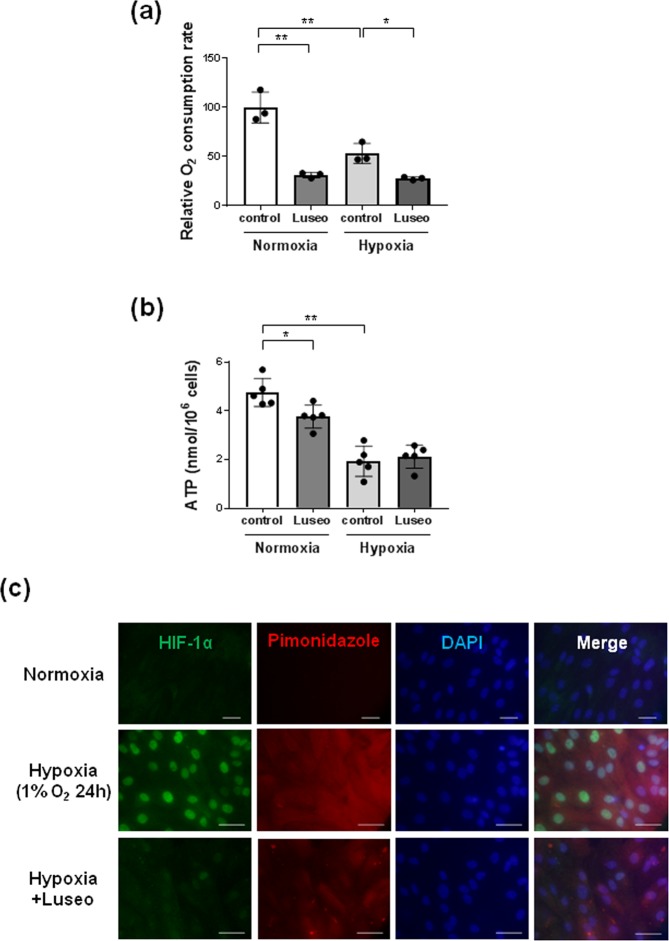
Luseogliflozin suppressed oxygen consumption and restored intracellular hypoxia in HRPTECs. (**a**) The oxygen consumption rate (OCR) of HRPTECs was measured as described in the methods. Luseogliflozin (100 µmol/l) inhibited the OCR in HRPTECs under normoxic conditions. Hypoxia significantly decreased the OCR, and luseogliflozin decreased the OCR, even under hypoxic conditions. All OCR levels are expressed as fold of control (n = 3). (**b**) Cell ATP levels during luseogliflozin treatment under normoxia and hypoxia. HRPTECs were treated with luseogliflozin for 24 h. At the end of the incubation, cells were extracted with perchloric acid for the measurement of ATP as described in the methods (n = 5). Hypoxia significantly decreased intracellular ATP, and luseogliflozin failed to decrease ATP under hypoxic conditions. All results are shown as the means ± SD. *p < 0.05, **p < 0.01, by one-way ANOVA followed by Tukey’s multiple comparison test. (**c**) Immunofluorescence analysis of HIF-1α and pimonidazole in HRPTECs. HRPTECs were grown on coverslides and then treated for 24 h. Hypoxia induced the nuclear expression of HIF-1α in HRPTECs, and luseogliflozin (100 µmol/l) inhibited hypoxia-induced HIF-1α expression. Hypoxia in HRPTECs was detected by pimonidazole hydrochloride. Luseogliflozin increased cellular oxygen levels in HRPTECs under hypoxic conditions. The nuclei were stained with DAPI. Scale bars, 30 µm for normoxia and 43.1 µm for hypoxia.

Because luseogliflozin has inhibitory effects on hypoxia-induced HIF-1α protein like the mitochondrial inhibitors (Fig. 2b), we examined the effects of luseogliflozin on mitochondrial respiration and ATP synthesis in HRPTECs. Luseogliflozin decreased the OCR under normoxic conditions to 31.3 ± 2.5% of that of the controls (*p* < 0.01) (Fig. 3a and Supplementary Table 5). Hypoxia also significantly decreased the OCR to 53.4 ± 10.4% of that of the controls in normoxia (*p* < 0.01), and luseogliflozin further inhibited the OCR under hypoxia to 52.5 ± 2.4% of that of the controls under hypoxia (*p* < 0.05) (Fig. 3a and Supplementary Table 5).

Luseogliflozin decreased intracellular ATP level to 79.2 ± 10.2% of that of the controls under normoxia (*p* < 0.05) (Fig. 3b and Supplementary Table 5). Hypoxia also significantly decreased the intracellular ATP level to 40.8 ± 13.2% of that of the controls under hypoxia (*p* < 0.05) (Fig. 3b and Supplementary Table 5). However, luseogliflozin failed to decrease ATP under hypoxic conditions (Fig. 3b and Supplementary Table 5).

### Luseogliflozin restores hypoxic conditions in HRPTECs

Because luseogliflozin decreased oxygen consumption in HRPTECs (Fig. 3a, Supplementary Table 5), we examined the effect of luseogliflozin on intracellular oxygen levels using the hypoxia-sensitive dye pimonidazole (Fig. 3c). Interestingly, luseogliflozin rescued the hypoxic state in HRPTECs, even under hypoxic conditions (Fig. 3c). Immunocytochemical analysis demonstrated that hypoxia apparently induced the nuclear expression of HIF-1α in HRPTECs, and luseogliflozin inhibited hypoxia-induced HIF-1α expression (Fig. 3c).

### Luseogliflozin improves hyperglycemia, but not blood pressure or albuminuria

To confirm the effects of luseogliflozin on proximal tubular cells *in vivo*, we treated type 2 diabetic *db/db* mice with luseogliflozin for 8 weeks. Diabetic *db/db* mice showed higher fasting blood glucose levels than lean control *db/m* mice (*p* < 0.01; Table 1), as well as HbA1c levels (*p* < 0.01; Table 1). Luseogliflozin failed to decrease the body weights of *db/db* mice (Table 1). Furthermore, luseogliflozin did not change the blood pressure in normotensive *db/db* mice (Table 1). No significant difference was observed in food intake among mice (Table 1). Compared with *db/m* mice, *db/db* mice showed polydipsia, and luseogliflozin decreased water intake in *db/db* mice (Table 1). In addition, luseogliflozin ameliorated polyuria in *db/db* mice, but this difference was not significant because of the wide variation (Table 1). *Db/db* mice had albuminuria (Table 1). Luseogliflozin monotherapy failed to ameliorate proteinuria in *db/db* mice as recently described^24^. In addition, *db/db* mice did not show significant changes in urinary and tissue KIM-1 levels compared with *db/m* mice as described in previous studies^25,26^, luseogliflozin tended to decrease KIM-1 levels in *db/db* mice (Table 1).

**Table 1:** Laboratory data of mice.

	*db/m*	*db/db*	*db/db* + Luseogliflozin
Fasting blood sugar (mmol/l)	3.11 ± 0.42	30.48 ± 4.50**	9.34 ± 0.79^††^
HbA1c (mmol/l)	15.83 ± 2.68	120.10 ± 10.54**	41.51 ± 2.89**^††^
HbA1c (%)	3.60 ± 0.24	12.93 ± 0.97**	5.95 ± 0.26**^††^
Food intake (g/day)	5.42 ± 1.22	6.61 ± 1.26	6.81 ± 0.77
Body weight (g)	27.51 ± 2.48	44.67 ± 6.80**	51.32 ± 1.60**
Mean blood pressure (mmHg)	57.04 ± 10.88	67.41 ± 10.33	72.80 ± 6.18
Water intake (ml/day)	4.90 ± 0.32	21.38 ± 3.63**	12.78 ± 1.37**^††^
Urinary volume (ml/day)	0.21 ± 0.16	8.33 ± 3.18**	7.13 ± 2.45**
Urinary albumin (μg/day)	4.15 ± 5.72	190.79 ± 159.75**	282.87 ± 104.02**
Urinary KIM-1 (pg/day)	187.08 ± 134.10	482.93 ± 422.46	188.32 ± 75.98
Tissue KIM-1 (pg/mg)	102.02 ± 19.95	89.03 ± 35.3	51.27 ± 15.85

Metabolic parameters and renal function of *db/m*, *db/db* mice and luseogliflozin-treated *db/db* mice. Eight-week-old *db/db* mice (n = 4) were treated with 15 mg/kg/day (0.01% in chow) luseogliflozin for 8 weeks. Urinary albumin and KIM-1 were log(e) transformed for parametric analysis. Values are means ± SD. *p < 0.05, **p < 0.01 vs *db/m* mice (n = 4). ^†^*p* < 0.05, ^††^*p* < 0.01 vs non-treated *db/db* mice (n = 5).

### Luseogliflozin attenuates HIF-1α and fibronectin expression in the renal cortex and ameliorates tubular injury in *db/db* mice

**Figure 4:**
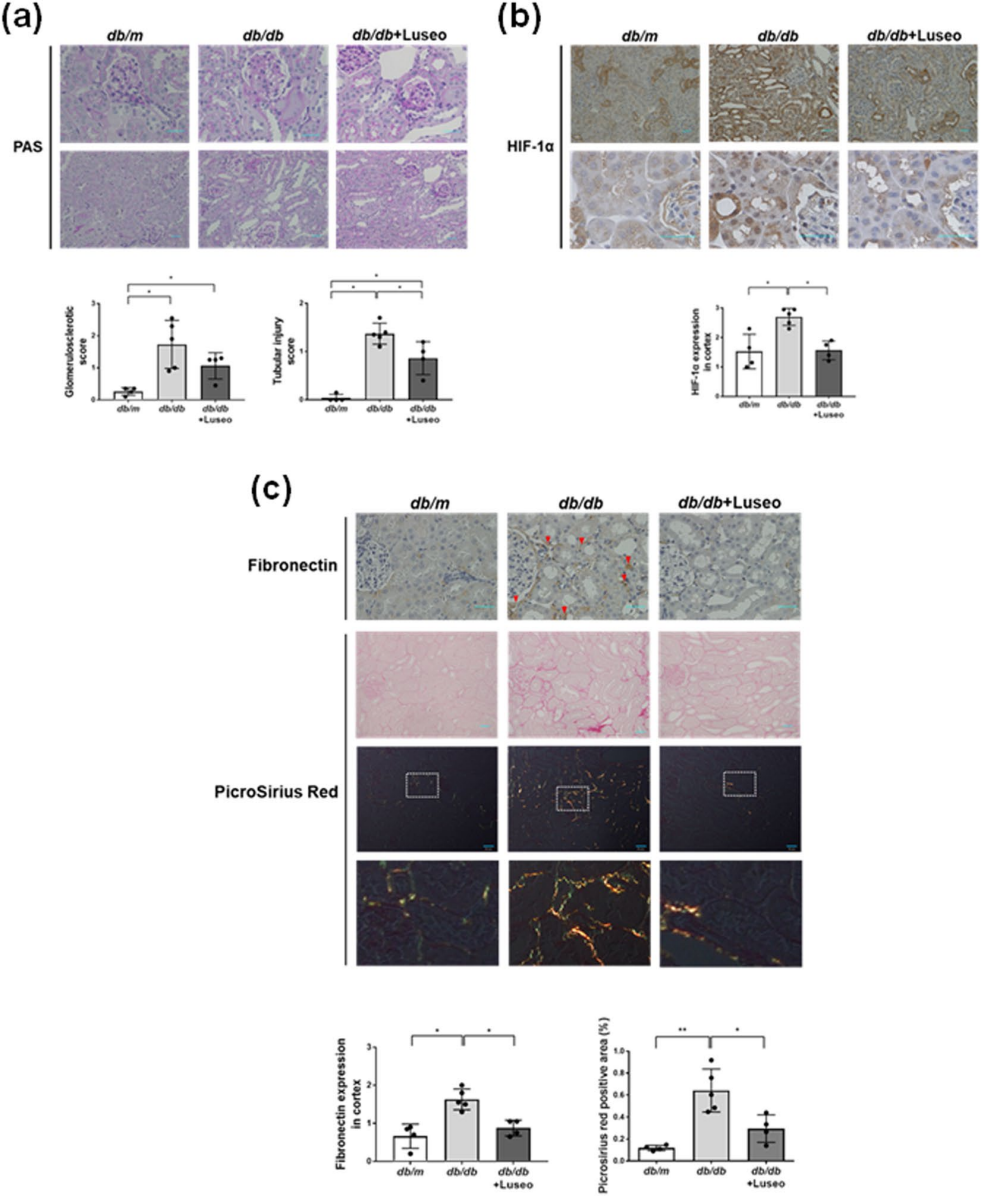
Luseogliflozin ameliorates tubular injury in *db/db* mice, accompanied by the inhibition of HIF-1α and tubulointerstitial fibrosis. (**a**) Periodic acid-Schiff (PAS) staining of the glomerular tuft area surrounded by the proximal tubules in each group of mice. Scale bars, 30 µm. (**b**) Immunohistochemistry for HIF-1 α protein. Scale bars, 30 µm. (**c**) Immunohistochemistry for fibronectin and Picrosirius Red staining. The red arrows show immunoreactive staining for fibronectin in *db/db* mice. Scale bars, 30 μm in the top and the second panels, and 50 μm in the middle panels. Bottom panels show higher magnification images of Picrosirius Red staining in the middle panels under polarized light. Data are semiquantitative morphometric analyses of the glomerulosclerotic score and tubular injury score (**a**), HIF-1α (**b**) and fibronectin expression (**c**). Comparisons by Kruskal-Wallis test followed by Man-Whitney *U* test for multiple comparisons. Picrosirius Red staining (**c**) was analyzed by one-way ANOVA, Tukey’s post hoc test. *p < 0.05, **p < 0.01. *Db/m* mice (n = 4), *db/db* mice (n = 5) and luseogliflozin-treated *db/db* mice (n = 4).

Luseogliflozin did not significantly improve glomerular sclerosis (Fig. 4a and Supplementary Table 6). However, compared to non-treatment, luseogliflozin significantly ameliorated tubular injury in *db/db* mice (*p* < 0.05) (Fig. 4a and Supplementary Table 6). The diabetic *db/db* mice showed strong nuclear HIF-1α expression in their cortical proximal tubules (Fig. 4b). Notably, luseogliflozin decreased positive immunostaining for HIF-1α and fibronectin and picrosirius red staining in the kidneys of *db/db* mice (Fig. 4b,c and Supplementary Table 6). A semiquantitative assessment of the immunohistochemistry results revealed that luseogliflozin significantly decreased positive staining for HIF-1α (Fig. 4b) in *db/db* mice, accompanied with the inhibition of fibronectin expression and Pirosirius Red staining (Fig. 4c) (*p* < 0.05) (Supplementary Table 6). There were no significant changes in HIF-1 target genes in kidney cortices of mice. However, *db/db* mice slightly increased the expressions of HIF-1 target genes compared with *db/m* mice (*Glut1*;1.00 ± 0.3 in *db/m* mice *vs*.1.30 ± 0.30 in *db/db* mice, *Pai1*;1.00 ± 0.47 in *db/m* mice *vs*.1.23 ± 0.22 in *db/db* mice, *p* > 0.05), and luseogliflozin tended to decrease these genes in *db/db* mice (Supplementary Table 7).

## Discussion

In the current study, we demonstrated that luseogliflozin inhibited hypoxia-induced nuclear HIF-1α expression and HIF-1 target genes in HRPTECs (Fig. 1a). In addition, we found that luseogliflozin decreased diabetes-induced HIF-1α expression in proximal tubular cells and tubulointerstitial injury in the renal cortex in *db/db* mice (Fig. 4a–c and Supplementary Table 6). This is the first study to demonstrate that an SGLT2 inhibitor suppresses the HIF-1α pathway in renal proximal tubular cells using *in vitro* and *in vivo* experiments.

In diabetic nephropathy, tubular injury is an important component of renal failure, and tubular hypoxia is a driving force for proximal tubulopathy^27^. Hyperglycemia induces glomerular hyperfiltration and increases tubular sodium and glucose reabsorption through SGLTs, which enhance sodium-potassium-ATPase activity, resulting in increased oxygen consumption. Thus, proximal tubular cells in the diabetic kidney are exposed to chronic hypoxia^28,29^.

Recently, several studies demonstrated that SGLT2 inhibitors ameliorated hypoxia in the kidney cortex in rodent models^30–32^. Acute SGLT inhibition by phlorizin, a dual inhibitor of SGLT1 and SGLT2, restored diabetes-induced reductions in renal cortex oxygen levels in streptozotocin (STZ)-induced diabetic Sprague-Dawley rats^30^. In addition, Layton *et al*. demonstrated that acute and chronic SGLT2 inhibition decreased sodium transport and oxygen consumption in an epithelial cell-based model of diabetic proximal tubules along a rat nephron^31^. Moreover, SGLT2 inhibitors significantly decreased pimonidazole immunostaining of the kidney cortex in a mouse model of ischemic reperfusion injury^32^.

**Figure 5:**
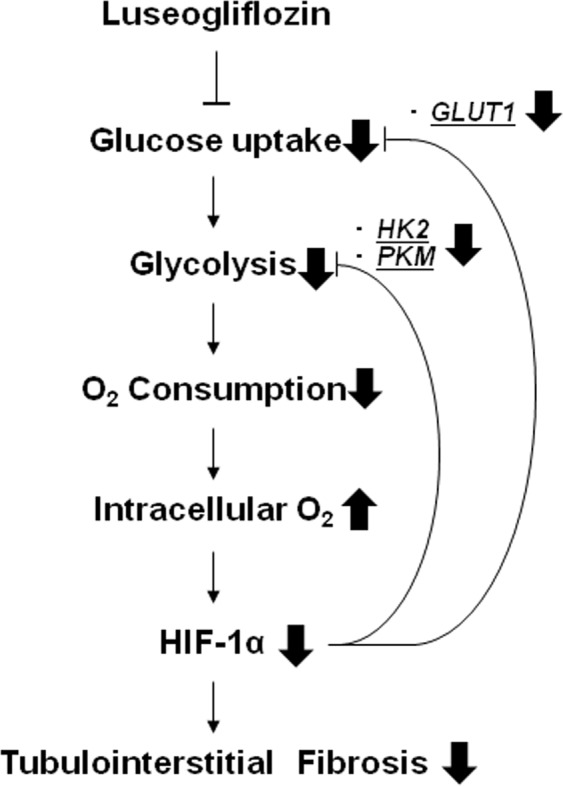
The renoprotective mechanism of the SGLT2 inhibitor occurs through oxygen metabolism in diabetic kidneys. Because luseogliflozin inhibits glucose uptake, which leads to subsequent glycolysis and mitochondrial respiration, luseogliflozin decreases oxygen consumption in renal proximal tubular cells. Subsequently, luseogliflozin-induced intracellular oxygen redistribution supplies oxygen for prolyl hydroxylase, which promotes HIF-1α degradation in the proteasome. Consequently, luseogliflozin inhibits hypoxia-induced HIF-1α protein expression and HIF-1-induced renal fibrosis in diabetic kidneys.

In this study, we found that luseogliflozin decreased the OCR and pimonidazole staining in HRPTECs even under hypoxic conditions (Fig. 3a,c). These data indicate that luseogliflozin inhibits HIF-1α expression through suppressing mitochondrial oxygen consumption, which leads to the restoration of intracellular hypoxia and subsequently promotes HIF-1α proteasomal degradation in HRPTECs (Fig. 5). These findings are similar to our previous report in which metformin, an antidiabetic agent, inhibited HIF-1α expression in the kidney cortex of Zucker diabetic fatty rats and HRPTECs by inhibiting mitochondrial respiratory function^33^.

In this study, we observed no change on cell viability of HRPTECs by the treatment with luseogliflozin despite of inhibition of SGLT2 and HIF-1-targeted GLUT1 under hypoxic conditions. Indeed, Biju *et al*. reported that the generation of adequate energy levels for the maintenance of renal proximal tubular cells viability under hypoxia does not require HIF-1, using HIF-1 deficient primary renal tubular epithelial cells^34^. Interestingly, they also showed that when glucose uptake or glycolysis was partially inhibited, the hypoxia-induced cell death and apoptosis onset was delayed in renal proximal tubular cells independent of HIF-1^34^. Renal proximal tubular cells are specialized to reabsorb the filtered of glucose from tubular fluid back into the blood^35^. Instead of glucose, lactate and glutamine were effective substrates for maintaining ATP levels in the renal proximal tubule^35^. Taken together with previous works, our results suggest that avoidance from hypoxia is more important in maintaining cell viability than glucose utilization via glucose transporters such as SGLT2 and GLUT1.

Furthermore, we found that luseogliflozin increased the phosphorylation of AMPKα in HRPTECs (Fig. 2a), in agreement with some studies^36–38^. In STZ-induced diabetic rats, phlorizin inhibited SGLT-coupled sodium-potassium-ATPase, which hydrolyzes ATP and activates AMPK through decreasing ATP/ADP and ATP/AMP ratios^36^. In addition, canagliflozin suppressed mitochondrial respiration by inhibiting mitochondrial complex I and increased AMPK phosphorylation in HEK-293 cells and mouse liver^37^ and in prostate and lung cancer cells^38^. Luseogliflozin decreased ATP levels concomitant with AMPK activation under normoxia (Fig. 2a and Fig. 3b). However, luseogliflozin failed to decrease ATP levels under hypoxia regardless of AMPK activation (Fig. 2a and Fig. 3b), indicating that luseogliflozin activates AMPK under hypoxia independent of any changes in the cellular ATP/ADP and ATP/AMP ratios. AMPK is activated by glucose deprivation^39^, but it is not confirmed whether this activation occurs via changes in ATP production. Recently, Zhang CS *et al*. found ATP-independent AMPK phosphorylation due to a decrease in the glycolytic metabolite fructose-1,6-bisphosphate (FBP)^40^. Luseogliflozin decreased HIF-1 target genes, such as the glucose transporter *GLUT1* and the glycolytic enzymes *HK2* and *PKM*, which have hypoxia response elements in their promoters^22,23^(Fig. 1b,e,f). Therefore, luseogliflozin inhibits glucose uptake and glycolysis in HRPTECs, which may result in a decrease in the glycolytic metabolite FBP, accompanied by AMPK activation, as described in a recent study^40^. These findings provide the underlying mechanism for luseogliflozin-induced AMPK activation independent of ATP under hypoxia.

Previous studies demonstrated that AMPK regulates HIF-1-mediated cellular metabolism^41,42^. However, our data showed that the AMPK activator AICAR failed to inhibit hypoxia-induced HIF-1α expression (Fig. 2b). These data imply that luseogliflozin-induced AMPK phosphorylation was not related to HIF-1α inhibition. Thus, our findings suggest that SGLT2 inhibitors may rescue renal proximal tubular cells from hypoxia and energy suppression by reducing oxygen and ATP consumption through inhibiting glucose entry following mitochondrial oxidative phosphorylation.

Although our results demonstrated that luseogliflozin improved pathological changes in the tubulointerstitial area in diabetic nephropathy, luseogliflozin failed to ameliorate albuminuria in *db/db* mice (Table 1). These data are consistent with those of a previous study^25^. Gallo *et al*. similarly demonstrated that empagliflozin failed to reduce albuminuria, urinary KIM-1 levels and glomerulosclerosis index in *db/db* mice^25^. In their study, *db/db* mice at 10 weeks of age were administered empagliflozin by oral gavage for 10 weeks^25^. The renoprotective effect of SLGT2 inhibitors in *db/db* mice might be highly dependent on the start time and duration of the treatment^43–46^. The earlier the treatment starts with SGLT2 inhibitor, the longer the treatment period and the greater the therapeutic effect on diabetic nephropathy. We recently clarified that luseogliflozin decreased the uptake of albumin in the proximal tubules of *db/db* mice by inhibiting cortical megalin expression, not by glomerular or tubular injury^24^. We demonstrated that *db/db* mice at 22-weeks old exhibited a significant decrease in the levels of the megalin protein in the kidneys accompanied with tubular injury^24^. Unexpectedly, luseogliflozin also decreased megalin expression in *db/db* mice with amelioration of tubutointerstitium fibrosis^24^. Furthermore, luseogliflozin decreased Texas Red conjugated-albumin uptake, suggesting that luseogliflozin induced albuminuria in *db/db* mice by inhibiting megalin expression^24^. Therefore, in the situation where albuminuria is used as the biomarker for diabetic nephropathy, we may overlook the renoprotective effect of SGLT2 inhibitors.

Recent basic experiments have already revealed that SGLT2 inhibitors ameliorated kidney fibrosis independent of their glucose-lowering effect in rodent models of chronic kidney diseases^36,47^. Further study is needed to confirm whether SGLT2 inhibitors ameliorate chronic hypoxia in the kidneys of diabetic and non-diabetic subjects.

In the current study, luseogliflozin ameliorated polydipsia but not polyuria, which might cause a chance of dehydration in *db/db* mice. SGLT2 inhibitors-induced reduction of body fluid could activate renin-angiotensin-aldosterone system (RAAS)^48^. Previous studies have shown that SGLT2 inhibition induces intrarenal RAS activity in *db/db* mice^25,49^ and increases circulating RAS mediators in patients with type1 diabetes^50,51^. RAAS inhibitors significantly provided a more favorable outcome in diabetic patients in EMPA-REG OUTCOME^52^. Thus, the beneficial therapeutic effects of SGLT2 inhibitors possibly need the combination therapy with RAS inhibitors.

In conclusion, we found that luseogliflozin, an SGLT2 inhibitor, ameliorated diabetic nephropathy at least partly by inhibiting HIF-1α accumulation. These data provide a novel mechanism for the renoprotective effects of SGLT2 inhibitors in diabetic nephropathy. Furthermore, this is the first study that SGLT2 inhibitor regulates the expression of HIF-1α, which could be implicated in the many hypoxic conditions such as cancer^53^, heart failure^54^, osteoporosis^55^ and amputation^56^ in diabetic patients. Clarifying the molecular regulatory mechanisms underlying HIF-1α expression by SGLT2 inhibitors could lead to the improvement to manage diabetes and other diabetic complications and comorbidities.

## Methods

### Materials and antibodies

Luseogliflozin was provided by Taisho Pharma, Co. (Tokyo, Japan). An anti-HIF-1α antibody was obtained from Novus Biologicals, Inc. (Littleton, CO, USA). Anti-AMP-activated protein kinase (AMPK)-α and anti-phosphorylated (p)-AMPKα (Thr 172) antibodies were obtained from Cell Signaling Technology, Inc. (Beverly, MA, USA). An anti-nucleoporin p62 antibody was obtained from BD Bioscience Japan, Inc. (Tokyo, Japan), and an anti-fibronectin antibody was obtained from Merck, Inc. (Kenilworth, NJ, USA). Alexa Fluor 594 donkey anti-mouse and Alexa Fluor 488 donkey anti-rabbit secondary antibodies were purchased from Invitrogen (Carlsbad, CA, USA). AICAR was purchased from Calbiochem (San Diego, CA, USA), and other chemicals and antibodies were obtained from Merck, Inc.

### Cell cultures

HPRTECs were purchased as once- or twice-passaged tubular cells from Lonza Walkersville, Inc. (Walkersville, MD, USA). The cells were grown in renal epithelial cell growth medium (REGM, Lonza) on collagen type 1-coated dishes at 37 °C in an incubator containing 5% CO2 and 95% humidified air as previously described^57^. Cells were exposed to reagents under normoxic (21% O_2_) or hypoxic (1% O_2_) conditions for 24 h and were then harvested for experiments as previously described^33^.

### Quantitative RT-PCR

Total RNA was extracted from HRPTECs and the cortex of right kidney from each group using an RNeasy mini kit (Qiagen, Tokyo, Japan) according to the manufacturer’s instructions. cDNA synthesis was performed with the SuperScript™ III First-Strand Synthesis System (Invitrogen, Carlsbad, CA, USA). Each cDNA sample was analyzed for gene expression by quantitative real-time PCR using a fluorescent TaqMan 57-nuclease assay and a sequence detection system (Prism 7300, Applied Biosystems, Carlsbad, CA, USA). TaqMan real-time PCR was performed using 2× TaqMan Master Mix and 20× assay-on-demand TaqMan primers and probes (Applied Biosystems). The analysis was performed with ABI Prism 7300 SDS software (Applied Biosystems). Unlabeled specific primers were purchased from Applied Biosystems for detecting the human glucose transporter 1 (*GLUT1*) gene (assay ID: Hs 00892681), human plasminogen activator inhibitor 1 (*PAI-1*) gene (assay ID: Hs 00167155), human vascular endothelial growth factor (*VEGF*) gene (assay ID: Hs 00900055), human hexokinase 2 (*HK2*) gene (assay ID: Hs 00606086), human pyruvate kinase M1/2 (*PKM*) gene (assay ID: Hs 00987254), human ribosomal protein lateral stalk subunit P0 (*RPLP0*) gene (assay ID: HS 00420895), mouse glucose transporter 1 (*Glut1*) gene (assay ID: Mm 00441480), mouse plasminogen activator inhibitor 1(*Pai1*) gene (assay ID: Mm 00435858) and mouse ribosomal protein lateral stalk subunit P0(*Rplp0*) gene (assay ID: Mm 00725448). After an initial 2 min at 50 °C and 10 min at 95 °C, the samples were cycled 55 times at 95 °C for 15 s and 60 °C for 1 min. For quantitative analysis, the cDNA content of each sample was normalized to the levels of the housekeeping gene *RPLP0* using the comparative CT method.

### Western blotting

Total cellular extracts and soluble nuclear extracts from HRPTECs were prepared as described previously^57,58^. Western blotting was carried out using 3–8% Novex NuPAGE Tris-acetate gels (Invitrogen) for HIF-1α and nucleoporin p62 or 4–12% NuPAGE Bis-Tris SDS-PAGE gels (Invitrogen) for p-AMPKα (Th172), AMPK and α-actinin under reducing conditions. After proteins were transferred onto a Hybond-P PVDF membrane (Amersham Biosciences Co., Piscataway, NJ, USA), the membranes were incubated with the primary antibodies (dilution 1:1000), incubated with a peroxidase-conjugated secondary antibody (dilution 1:50000) (Amersham), and visualized with an enhanced chemiluminescence (ECL) system (Amersham). Selected blots were washed and reprobed with an antibody against nucleoporin p62 for nuclear protein extracts and α-actinin for total cellular extracts to control for small variations in protein loading and transfer. Images were processed using ImageJ (U. S. National Institutes of Health, Bethesda, MD, USA) for densitometric analysis. Signal intensities in the control lanes were arbitrarily assigned a value of 1.00.

### Oxygen consumption rate (OCR) measurements

OCRs were measured using an oxygen consumption assay (Agilent, Santa Clara, CA, USA) as described in a previous study^59^. Briefly, HRPTECs were cultured on black, clear bottom, collagen type 1-coated 96-well plates (Corning, NY, USA). After serum starvation for 24 h with serum-free DMEM, cells were exposed to normoxic or hypoxic conditions for 24 h. Subsequently, phosphorescent oxygen-sensitive probes were added to the culture medium with or without 100 µmol/l luseogliflozin, and the plates were measured with a fluorescent plate reader using a time-resolved fluorescence method (EnSpire, PerkinElmer Japan, Tokyo, Japan). Culture medium was changed from serum-free DMEM to REGM during the OCR measurement.

### Intracellular ATP measurements

ATP amounts were measured using an ATP assay kit (Abcam, Cambridge, UK) according to the manufacturer’s instructions^60^. HRPTECs were cultured on collagen type 1-coated dishes as described above. Then, the cells were lysed in ATP assay buffer and deproteinized with trichloroacetic acid (TCA) (Abcam). Intracellular ATP was measured by a GloMax Discover microplate plate reader (Promega, Madison, WI, USA).

### Immunocytofluorescence

Immunocytofluorescence was performed as described previously^33^. HRPTECs were cultured on collagen type 1-coated four-chamber glass slides (BD Biosciences). After exposure to 100 µmol/l luseogliflozin for 24 h under normoxic or hypoxic conditions, the cells were fixed with 100% ethanol for 10 min and incubated with a rabbit polyclonal anti-HIF-1α antibody (1:200) at room temperature for 1 h. Then, the cells were rinsed with PBS and subsequently incubated with an Alexa Fluor 488 donkey anti-rabbit secondary antibody (Invitrogen) at 1:500 dilution for 1 h at room temperature. Finally, the slides were analyzed by confocal laser scanning microscopy.

### Detection of cellular hypoxia

Cellular hypoxia was detected by adding pimonidazole hydrochloride (200 mmol/L; Hypoxyprobe-1, Hydroxyprobe. Inc., Burlington, MA), which binds to cells or tissues with pO_2_ levels below 10 mmHg, to HRPTECs that were treated with 100 µmol/l luseogliflozin and exposed to hypoxia (1% O_2_) for 24 h. Staining was performed according to the manufacturer’s instructions using an Alexa Fluor 594 donkey anti-mouse secondary antibody as previously described^33^.

### Animals

All animal experiments followed the National Institutes of Health Guide for the Care and Use of Laboratory Animals and were approved by the Research Center for Animal Life Science of Asahikawa Medical University. We purchased male *db/m* mice (on a C57BLKs/J background) and *db/db* (Lepr^*db/db*^) mice from CLEA Japan, Inc. (Tokyo, Japan). Animals purchased at 7 weeks of age were housed on a 12-hr light/dark cycle and provided regular chow (MF, Oriental Yeast Co., Tokyo, Japan) ad libitum and tap water. Diabetic *db/db* mice were randomly assigned to two groups at the age of 8 weeks. A first group did not receive an active pharmacological treatment and were used as a control. A second group received luseogliflozin (0.01% in chow; 15 mg/kg body weight/day) in a regular rodent diet (MF) for 8 weeks to examine the effects of an SGLT2 inhibitor on the diabetic kidney. No adverse effects were founded in luseogliflozin-treated *db/db* mice. Systolic blood pressure (SBP) and mean blood pressure (MBP) were measured in conscious mice using an automated tail-cuff manometer system (MK-2000ST; Muromachi Kikai, Tokyo, Japan). The average of 10 consecutive measurements from each mouse was calculated. Glucose levels in whole blood extracted from the tail were quantified using a One Touch glucose analyzer (LifeScan Inc., Milpitas, CA, USA). HbA1c levels were measured using a DCA 2000 analyzer (Siemens Medical Solutions Diagnostics, Tokyo). Mice in each group were placed in metabolic balance cages for 24-h urine collection. Renal function was assessed by measuring urinary albumin excretion (UAE) (Exocell, Philadelphia, PA, USA) and urinary kidney injury molecule-1 (KIM-1) and KIM-1 in kidney tissue (Abcam) using ELIZA kits.

### Morphological analysis and immunohistochemistry

Glomerulosclerotic scores were evaluated by a semiquantitative method in 20 glomeruli per animal using 2-μm kidney sections stained with periodic acid-Schiff (PAS) stain^61^. Assessment of tubulointerstitial injury was evaluated in the cortical regions using PAS staining and a semiquantitative scoring system evaluating interstitial fibrosis, inflammation, tubular atrophy, tubular dilation, debris accumulation, and cast formation in 20 tubulointerstitial areas per animal. A score of (0) for normal tubulointerstitium, (1) for injury in less than 25%, (2) for injury up to 50%, and (3) for injury in more than 50% of the biopsy specimen, as described in the previous study^33^. Immunohistochemistry was performed with a rabbit polyclonal anti-HIF-1α antibody (1:200) (Novus Biologicals) and mouse monoclonal anti-fibronectin antibody (1:500) (Santa Cruz Biotechnology, Santa Cruz, CA, USA) as previously described^33^. The picrosirius red stain was performed by Picrosirius Red Stain Kit (Polysciences, Warrington, PA, USA) and evaluated by optical microscope and polarizing microscope. The picrosirius red-positive area was measured using Image J by identifying the percentage of interstitial collagen positive region at x 32 magnification in five randomly selected regions^62^. Morphometry was conducted in a blinded manner by two experienced nephrologists.

### Statistical analysis

The sample sizes for the animal studies were determined according to a previous publication^24^. At least three separate experiments were performed per protocol. Each treatment group was assayed in duplicate for real-time RT-PCR and OCR assays. The values shown represent the means ± standard deviation (SD). All measured parametric variables were log(e) transformed for all statistical analyses. For parametric tests, statistical analysis was performed by ANOVA and Tukey’s post hoc analysis. Non-parametric analyses of histological scores were conducted using a Kruskal-Wallis test with the unpaired, non-parametric Mann-Whitney U test as a post hoc analysis. Values of P < 0.05 were considered statistically significant. ANOVA and Tukey’s post hoc analysis were performed using GraphPad Prism ver. 7.0 software (San Diego, CA, USA). The other statistical analyses described above were performed using SPSS ver. 24 (Chicago, IL, USA).

## Supplementary information

Supplementary Tables
