# Supplementary material for: Hypoxia-inducible factor-1α is the therapeutic target of the SGLT2 inhibitor for diabetic nephropathy
Source: Sci Rep. 2019 Oct 14;9:14754. doi: 10.1038/s41598-019-51343-1 (PMC6791873; doi:10.1038/s41598-019-51343-1)
Supplement: Supplementary file 1 — Supplementary Tables [file 41598_2019_51343_MOESM1_ESM.docx]

**Supplementary Tables**

**Hypoxia-inducible factor-1α is the therapeutic target of the SGLT2 inhibitor for diabetic nephropathy**

Ryoichi Bessho, Yumi Takiyama, Takao Takiyama, Hiroya Kitsunai, Yasutaka Takeda, Hidemitsu Sakagami and Tsuguhito Ota

Division of Metabolism and Biosystemic Science, Department of Internal Medicine, Asahikawa Medical University, Japan

**Corresponding author:**

**Corresponding authors:** Yumi Takiyama, Division of Metabolism and Biosystemic Science, Department of Internal Medicine, Asahikawa Medical University, 2-1-1-1, Midorigaoka Higashi, Asahikawa 078-8510, Japan. Phone: +81-166-68-2454; E-mail: taka0716@asahikawa-med.ac.jp; ORCID iD: https://orcid.org/0000-0002-7443-7817; and Tsuguhito Ota, E-mail: ota@asahikawa-med.ac.jp; ORCID iD:　https://orcid.org/0000-0002-2216-8459.

.

**Supplementary Table 1. Effects of luseogliflozin on the hypoxia-induced HIF-1α protein expressions.**

|  | **Normoxia** | **Hypoxia** | | | |
| --- | --- | --- | --- | --- | --- |
|  | **Control** | **Control** | **Luseo 1 µmol/l** | **Luseo 10 µmol/l** | **Luseo 100 µmol/l** |
| **HIF-1α/p62** | 1.00±0.00 | 8.41±0.34** | 7.02±0.82 | 6.05±1.16† | 5.95±0.19† |

Western blot analysis of HIF-1α protein expression in HRPTECs. The density of the bands is quantified by Image J as described in Method. Luseogliflozin inhibited hypoxia-induced HIF-1α protein expression. Values are means ± SD (*n*=3). * *p*<0.05, ** *p*<0.01 vs normoxic control. † *p*<0.05, †† *p*<0.01 vs hypoxic control.

**Supplementary Table 2. Effects of luseogliflozin on the hypoxia-induced HIF-1α target gene expressions.**

|  | | **Normoxia** | | | **Hypoxia** | |
| --- | --- | --- | --- | --- | --- | --- |
|  | **Control** | | **Luseogliflozin** | **Control** | | **Luseogliflozin** |
| ***GLUT1/RPLP0*** | 1.00±0.00 | | 0.69±0.03 | 27.73±2.88** | | 20.90±1.84**†† |
| ***PAI-1/RPLP0*** | 1.00±0.00 | | 1.02±0.09 | 67.50±3.81** | | 46.41±3.42**†† |
| ***VEGF/RPLP0*** | 1.00±0.00 | | 1.22±0.35 | 27.73±1.03** | | 21.36±1.49**†† |
| ***HK2/RPLP0*** | 1.00±0.00 | | 0.67±0.05 | 19.73±0.98** | | 13.97±1.17**†† |
| ***PKM/RPLP0*** | 1.00±0.00 | | 0.98±0.05 | 1.43±0.10* | | 1.24±0.05† |

Quantitative real-time RT-PCR analysis of HIF-1 target genes. The relative amounts of *GLUT1*, *PAI-1*, *VEGF*, *HK2* and *PKM* mRNA were normalized by *RPLP0* and expressed as an arbitrary unit in which the control group value equaled 1. Luseogliflozin (100 µmol/l) inhibited hypoxia-induced HIF-1α target gene expressions. Values are means ± SD (*n*=3). * *p*<0.05, ** *p*<0.01 vs normoxic control. † *p*<0.05, †† *p*<0.01 vs hypoxic control.

**Supplementary Table 3.** **Effects of luseogliflozin on the AMPK phosphorylation.**

|  | | **Normoxia** | | | **Hypoxia** | |
| --- | --- | --- | --- | --- | --- | --- |
|  | **Control** | | **Luseogliflozin** | **Control** | | **Luseogliflozin** |
| **pAMPKα /AMPKα** | 1.00±0.00 | | 1.99±0.52* | 1.05±0.22 | | 1.90±0.20† |

Western blot analysis of pAMPKα and AMPKα protein expressions in HRPTECs. The density of the bands is quantified by Image J as described in method. Luseogliflozin (100 µmol/l) promoted phospholylations of AMPK in normoxia and hypoxia. Values are means ± SD (*n*=3). * *p*<0.05 vs normoxic control. † *p*<0.05 vs hypoxic control.

**Supplementary Table 4. Effects of AMPK activator, inhibitor and mitochondrial inhibitors on hypoxia-induced HIF-1α expression.**

|  | **Normoxia** | **Hypoxia** | | | | | |
| --- | --- | --- | --- | --- | --- | --- | --- |
|  | **Control** | **Control** | **Luseogliflozin** | **AICAR** | **Compound C** | **Rotenone** | **Antimycycin A** |
| **HIF-1α/p62** | 1.00 ± 0.00 | 5.30 ± 0.38** | 3.72 ± 0.85† | 4.68 ± 0.69 | 5.92 ± 0.80 | 0.41 ± 0.26†† | 0.56 ± 0.37†† |

Western blot analysis of HIF-1α protein expressions in HRPTECs. The density of the bands is quantified by Image J as described in method. Luseogliflozin (100 µmol/l) inhibited hypoxia-induced HIF-1α protein expression. The AMPK activator, AICAR (1 mmol/l) and the AMPK inhibitor Compound C (20 µmol/l) failed to suppress hypoxia-induced HIF-1α expression. The inhibitors for mitochondrial respiratory complexes I (1 µmol/l Rotenone) and III (10 ng/ml Antimycin A) inhibited hypoxia-induced HIF-1α accumulation. Values are means ± SD (*n*=3). ** *p*<0.01 vs normoxic control. † *p*<0.05, †† *p*<0.01 vs hypoxic control.

**Supplementary Table 5. Effects of luseogliflozin on mitochondrial functions.**

|  | **Normoxia** | | **Hypoxia** | |
| --- | --- | --- | --- | --- |
|  | **Control** | **Luseogliflozin** | **Control** | **Luseogliflozin** |
| **Relative OCR (*n*=3)** | 100.00 ± 15.90 | 31.30 ± 2.50** | 53.40 ± 10.40** | 27.50 ±1.30† |
| **ATP (nmol/10^6^ cells) (*n*=5)** | 4.76 ± 0.57 | 3.77 ± 0.48* | 1.94 ± 0.62** | 2.12 ± 0.48 |

Relative oxygen consumption rate (OCR) levels and measured ATP levels in HRPTECs. Values are means ± SD (*n*=3). * *p*<0.05, ** *p*<0.01 vs normoxic control. † *p*<0.05 vs hypoxic control. Luseogliflozin (100 µmol/l) inhibited OCR in HRPTECs in normoxia. Hypoxia significantly decreased OCR, and luseogliflozin decreased OCR under hypoxic condition. In addition, luseogliflozin (100 µmol/l) decreased cell ATP levels under normoxia. Hypoxia significantly decreased intracellular ATP, and luseogliflozin fail to decrease ATP under hypoxic condition.

**Supplementary Table 6. The semiquantitative morphometric analysis and immunoreactive scores.**

|  | ***db/m*** | ***db/db*** | ***db/db*+Luseogliflozin** |
| --- | --- | --- | --- |
|  | (n=4) | (n=5) | (n=4) |
| **Gromerulosclerotic score** | 0.26 ± 0.12 | 1.73 ± 0.75* | 1.06 ± 0.41* |
| **Tubular injury score** | 0.04 ± 0.08 | 1.37 ± 0.22* | 0.86 ± 0.34*† |
| **HIF-1α immunoreactivity** | 1.53 ± 0.59 | 2.70 ± 0.29* | 1.56 ± 0.32† |
| **Fibronectin immunoreactivity** | 0.66 ± 0.32 | 1.63 ± 0.27* | 0.88 ± 0.21† |
| **PicroSirius Red Stain** | 0.12 ± 0.02 | 0.64 ± 0.16** | 0.29 ± 0.13† |

Semiquantitative morphometric analysis and immunoreactive scores in mice. Scores were evaluated by a semi-quantitative method in 20 glomeruli or tubulointerstitial areas per animal as described in method. Luseogliflozin significantly decreased tubular injury score and immunoreactive scores of HIF-1α and fibronectin, and PicroSirius Red staining in the renal cortex. Values are means ± SD. * *p*<0.05 vs *db/m* mice. † *p*<0.05 vs non-treated *db/db* mice

**Supplementary Table 7. Effects of luseogliflozin on HIF-1 target genes in the kidneys of mice.**

|  | ***db/m*** | ***db/db*** | ***db/db*+Luseogliflozin** |
| --- | --- | --- | --- |
|  | (n=4) | (n=5) | (n=4) |
| ***Glut1/Rplp0*** | 1.00 ± 0.30 | 1.30 ± 0.30 | 0.92 ± 0.18 |
| ***Pai-1/Rplp0*** | 1.00 ± 0.47 | 1.23 ± 0.22 | 1.15 ± 0.45 |

Quantitative real-time RT-PCR analysis of HIF-1 target genes in the kidneys of mice. The relative amounts of *Glut1*, *Pai-1* mRNA were normalized by *Rplp0* and expressed as an arbitrary unit in which the *db/m* group value equaled 1. Values are means ± SD. HIF-1-targeted genes in the cortex of the kidneys did not significantly increase in *db/db* mice. In addition, *db/db* mice treated with luseogliflozin failed to decrease HIF-1-targeted genes compared with those in *db/db* mice, because of wide variations among mice.

.
